# Immature Oocyte for Fertility Preservation

**DOI:** 10.3389/fendo.2019.00464

**Published:** 2019-07-17

**Authors:** Weon-Young Son, Sara Henderson, Yoni Cohen, Michael Dahan, William Buckett

**Affiliations:** Department of Obstetrics and Gynecology, MUHC Reproductive Centre, McGill University Health Center, McGill University, Montréal, QC, Canada

**Keywords:** cancer, fertility preservation, immature oocyte, *in vitro* maturation, vitrification

## Abstract

*In vitro* maturation (IVM) of human immature oocytes has been offered to women who are at risk of developing ovarian hyperstimulation syndrome (OHSS) caused by gonadotropin stimulation, such as PCO(S) patients or who have poor ovarian reserve. Cryopreservation of oocytes matured *in vivo* obtained in IVF cycles has improved after implementing the vitrification method and many successful results have been reported. Now, this procedure can be successfully offered to fertility preservation programs for patients who are in danger of losing their ovarian function due to medical or social reasons, and to oocyte donation programs. This vitrification technique has also been applied to cryopreserve oocytes obtained from IVM program. Some advantages of oocytes vitrification related with IVM are: (1) eliminating costly drugs and frequent monitoring; (2) completing treatment within 2 to 10 days (3) avoiding the use of hormones in cancer patients with hormone-sensitive tumors; and (4) retrieving oocytes at any point in menstrual cycle, even in the luteal phase. In addition, immature oocytes can also be collected from extracorporeal ovarian biopsy specimens or ovaries during caesarian section. Theoretically, there are two possible approaches for preserving immature oocytes: oocyte cryopreservation at the mature stage (after IVM) and oocyte cryopreservation at the Germinal Vesicle (GV)-stage (before IVM). Both vitrification of immature oocyte before/after IVM is not currently satisfactory. Nevertheless, many IVF centers worldwide are doing IVM oocyte cryopreservation as one of the options to preserve fertility for female cancer. Therefore, more studies are urgently required to improve IVM- and vitrification method to successfully preserve oocytes collected from cancer patients. In this review, present oocyte maturation mechanisms and recent progress of human IVM cycles will be discussed first, followed by some studies of the vitrification of human IVM oocyte.

## Introduction

Fertility preservation is a technique which may prolong the ability to conceive, either for medical or social reasons. Cancer is a major health concern, though survival rates have increased as treatment methods are improving, especially in young people (1). However, treatment of these cancers is commonly detrimental to reproductive function, with a high risk of losing one's fertility even after recovery, especially for young cancer survivors (2, 3).

Today, it is possible to preserve reproductive potential for these cancer patients using cryopreservation technic of either embryos, oocytes, or ovarian tissue (4). Embryo cryopreservation is a well-established routine procedure in IVF clinics worldwide and is the option with the best chances of reproductive success in the future that is offered to female cancer patients (5). However, since it requires a sperm source, many young single women are not able to choose this option unless they want to use donor sperm. In addition, there may be other various constraints to produce embryos and subsequently store them due to ethical, religious and social reasons (6).

In these cases, oocyte cryopreservation is an alternative option to embryo freezing (6). Although the first live birth using slow-cooling method was reported in 1986 (7), it remains technically challenging and has yet to become a routine procedure in IVF laboratories until oocyte vitrification method is properly established (8, 9).

Oocyte vitrification now offers increased success rates in comparison to slow freezing worldwide. Several IVF centers in the world have reported similar pregnancy rates between fresh- and vitrified-oocytes matured *in vivo* obtained in IVF cycles (10–13). The American Society of Reproductive Medicine (ASRM) and American Society of Clinical Oncology (ASCO) have endorsed oocyte cryopreservation as a “fertility preservation strategy for women with cancer and other illnesses requiring treatments that pose a serious threat to their future fertility” (14, 15). Nevertheless, oocyte cryopreservation requires ovarian stimulation, thus potentially resulting in a delay in cancer treatment.

Other fertility preservation methods include ovarian tissue cryopreservation and *in-vitro* maturation (IVM). Both methods can be performed without a delay in cancer treatment even for prepubertal girls. Ovarian tissue cryopreservation method requires two surgical procedures such as harvesting and orthotopic transplantation of the tissues after thawing (16). There is also the risk of reintroducing malignant cells at transplantation (16, 17).

IVM oocyte cryopreservation involves the retrieval of immature oocytes from ovaries after minimal or no gonadotropin priming and then either cryopreservation at immature stage or at matured stage after IVM. There are several advantages of IVM such as simplified treatment, reduced cost and avoidance of potential side effects such as ovarian hyperstimulation syndrome (OHSS). IVM program has already been offered to women with polycystic ovary syndrome (PCOS) to avoid the risk of developing OHSS caused by exogenous gonadotrophin stimulation. Although the clinical outcome is still suboptimal, improved pregnancy rates in IVM cycles of PCO(S) patients have recently been reported by some centers to be 32.4 and 46.7% clinical pregnancy per embryo transfer (18, 19). Based on these results, currently, IVM technique has also been applied for women with poor response to ovarian stimulation (20, 21) and for women who need fertility preservation urgently (22–24). However, IVM procedure is still considered as experimental (25) and small number of IVF centers are doing this procedure worldwide since antagonist cycle, GnRH agonist triggering, and elective cryopreservation strategies have been improved.

**Figure 1:**
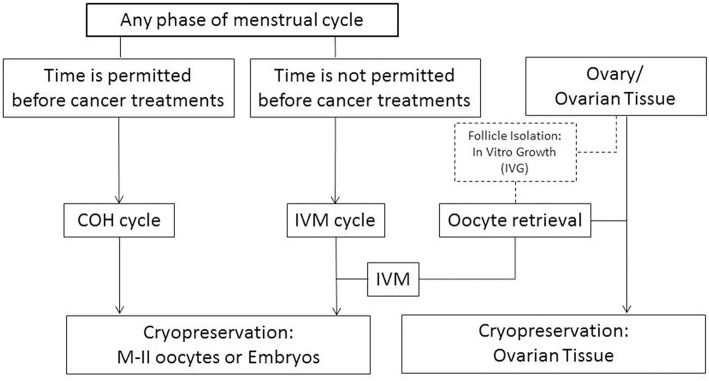
Fertility preservation strategies for female cancer patients depending on their clinical situations. COH, controlled ovarian hyperstimulation; IVM, *in vitro* maturation. Discontinuous lines/box: experimental procedure.

Although vitrification of oocytes retrieved from IVF cycles has been used successfully in the oocyte donation and fertility preservation programs, controlled ovarian stimulation for IVF is contraindicated for patients with certain forms of cancer. In addition, many cancer patients do not have enough time to do an IVF cycle before beginning chemo- or radiation-therapy. In these cases, immature oocyte collection can be an alternative (Figure 1) (26). However, very few live births have been reported after IVM oocyte cryopreservation. The first live birth was reported after oocyte cryopreservation using the slow-cooling method at the immature Germinal Vesicle (GV)-stage oocytes retrieved from conventional IVF cycles (27). Following this, the McGill Reproductive Center reported 5 pregnancies with live births after vitrification at MII-stage after IVM of immature oocytes collected from hCG-primed IVM cycles (28). However, as of yet, there have been no reports of successful pregnancies or live births after cryopreservation of IVM oocytes using either slow-cooling or vitrification method for cancer patients.

Therefore, it is important to understand oocyte maturation mechanisms and the current status of the human IVM program in order to make improvements in this program and in IVM oocyte vitrification procedures. In this manuscript, we will review our understanding of oocyte maturation mechanisms and recent advances in the field of human IVM cycles. Following this, some studies of the vitrification of human IVM oocytes will be discussed.

## Methods

This review is based on material published found via an electronic search of PUBMED between January 1996 and January 2019. We included articles that were published in English language for studies regarding fresh and cryopreserved oocytes produced from IVM program.

### Oocyte Maturation

It is important to understand the mechanism of oocyte maturation *in vivo* in order to obtain “high quality oocytes” *in vitro*. Oocyte maturation consists of nuclear maturation and cytoplasmic maturation (29). Oocyte nuclear maturation implies the re-initiation of the first meiotic division and progression to metaphase two. This process can be divided into several parts including meiotic resumption/germinal vesicle breakdown (GVBD), chromatin condensation, formation of the meiotic spindle, separation of chromosome with extrusion of first polar body, and meiotic re-arrest before fertilization (29). Cytoplasmic maturation involves metabolic structural changes in the organelle that will lead to successful fertilization and early embryo development (29).

Oocyte maturation *in vivo* is a complex process regulated through hormonal signals, interactions with surrounding somatic cells, and involvement of transcription factors regulating gene expression (30). It has been established that meiotic arrest until luteinizing hormone (LH) surge is regulated by cyclic adenosine 3', 5'-monophosphate (cAMP) levels within the oocyte. The high level of cAMP is mainly controlled by gap junction connection between oocytes and cumulus cells (CC), and between CC themselves (30). Each gap junction is composed of connexin (Cx) proteins such as Cx37 and Cx43 (30). *In vivo*, there are three mechanisms to maintain the high cAMP level within the oocyte before the LH surge. (1) cAMP enters the egg from CC through the gap junction (31). (2) cAMP is produced by the oocyte itself via G-protein coupled receptors in the oocyte membrane (32–34). (3) Guanosine 3′,5′-cyclic monophosphate (cGMP), which is produced in the mural and cumulus granulosa cells via the activity of the guanylate cyclase natriuretic peptide receptor 2 (NPR2) by C-type natriuretic peptide (CNP), passes through gap junctions into the oocyte (35–39), where it inhibits hydrolysis of cAMP by the phosphodiesterase 3A (PDE3A:oocyte specific phosphodiesterase) (40). The high intra-oocyte cAMP concentration inactivates meiosis promoting factor and thus blocks meiotic progression.

After the LH surge, oocyte maturation is induced by cascade signaling pathways as well as physiological changes in preovulatory follicles *in vivo*. Mural granulosa layers express LH-receptors (LH-R) in much higher numbers than those in CC. Therefore, LH activation of mural granulosa cells induces the expression of the EGF-like growth factors such as betacellulin, amphiregulin, and epiregulin, as second signals (41, 42). The EGFs bind to their receptors in cumulus cell and mitogen-activated protein kinase (MAPK) in CC is activated immediately. The increased activation of MAPK may achieve meiosis resumption by inducing synthesis of downstream meiosis resumption-inducing factor(s) as well as blocking the gap junction via phosphorylation of gap junction proteins. In addition, the LH surge inactivates NPR2 and activates cGMP phosphodiesterase PDE5, and induces a rapid drop in follicular and oocyte cGMP levels resulting in decreasing cGMP supply to the oocyte (37, 38, 43, 44). Physiologically, a Graafian follicle rapidly increases in volume by inflow and accumulation of follicular fluid. Concomitantly, the cumulus oocyte complex (COC) synthesizes a muco-elastic extracellular matrix (ECM). This brings about volumetric enlargement called cumulus expansion which is important to oocyte. Hyaluronan (HA) is mainly involved in the cumulus cell expansion after the LH surge *in vivo* and HA is synthesized by HAS2 (Hyaluronan synthase) in the plasma membrane and directly extends into the ECM (45–47). The gap junctions in the COCs are disrupted by the cumulus cell expansion which stops the transport of cAMP through the gap junction, and leads to activation of MPF and meiotic resumption of oocytes (48, 49). Oocyte itself is also actively involved in HA synthesis related with cumulus expansion while secreting soluble factors. Growth differentiation factor-9 (GDF-9), bone morphogenetic protein 15 (BMP-15), and BMP-6 are likely candidate molecules for oocyte-secreted factors (50, 51). These growth factors induce HAS2 gene expression and cumulus expansion in the presence of FSH (50, 52).

#### Clinical Application of Human IVM Program

There are differences in the process of oocyte maturation *in vivo* and *in vitro*. *In vivo*, although a follicle gets dominance and grows to Graafian follicle, the fully grown oocyte inside of the follicle remains arrested in GV-stage (prophase I) until LH surge (53). However, the immature oocytes retrieved from small antral follicles start nuclear maturation spontaneously *in vitro* (53). This spontaneous maturation causes a premature breakdown of oocyte-cumulus cell gap junctions, leading to a loss of beneficial cumulus cell metabolites, such as mRNA, proteins, substrates, and nutrients that are required to achieve successful fertilization and embryo developmental competence (53). Researchers think that is the main reason why IVM oocytes have lower reproductive potential than those of IVF oocytes in general (53). Therefore, some studies have tried to mimic *in vivo* systems by the approach of delaying or temporarily preventing spontaneous IVM with CNP (54) or with chemicals such as cAMP analog, kinase or PDE inhibitors (53, 55–58). However, the effect is still unknown and more research is required.

In the human IVM program, *in vivo* stimulation with gonadotropins has been commonly used to improve the quantity and quality of oocytes such as FSH-, hCG-, and combined FSH-hCG-primed IVM cycles (59). In IVM cycles at McGill, hCG-priming is performed prior to egg collection in IVM cycles.

The hCG-primed IVM program has improved following several studies related to both clinical and embryological aspects over the last several years (60).

#### Clinical Protocol

In the routine IVM procedure, a baseline ultrasound scan (US) is performed for all patients between Days 2 and 4 of the menstrual cycle to confirm that no ovarian cysts are present and to assess the antral follicle count (AFC). A transvaginal US is repeated on Days 8–12 of the cycle until the dominant follicle reaches ≤12 mm, and the endometrial thickness is at least 6 mm, after which 10,000 IU hCG is administered.

In the case of fertility preservation for cancer patients, immature oocyte retrieval is performed at any point in the menstrual cycle depending on timing of chemotherapy, either in the early-, late-follicular, or luteal stage when hCG priming was performed (22).

Oocytes retrieval is performed 38 h after hCG administration with a specially designed 19-gauge single-lumen aspiration needle (K-OPS-7035-RWH-ET; Cook, Australia). The aspiration pressure is 85 mmHg.

#### Laboratory Procedures

Laboratory process of IVM cycles is more time consuming and technically demanding than IVF cycles (60). Therefore, the embryologists should obtain adequate training from an individual skilled in this technique. It is important also to define the best conditions for the laboratory procedures in IVM cycles in order to increase clinical outcomes.

At the time of collection, the follicular aspirate is first examined under a stereomicroscope to identify COC. After, the follicular aspirate is filtered through a cell strainer composed of nylon mesh with 70-μm pores to identify more oocyte with few CC.

In the IVM cycles treated with hCG priming, *in vivo* mature oocytes may be retrieved at oocyte collection (60). In the IVM cycles without hCG priming, no *in vivo* mature oocytes can be retrieved at collection or on the day of retrieval.

The complex culture media as the basic IVM media have been used in research or in clinical purposes of human immature oocyte culture for IVM (60). Recently, commercialized IVM media such as SAGE (Coopersurgical) IVM medium and Medi-Cult IVM medium have been used in several IVF centers as they have the advantage of being certified as IVF quality controlled (61–63). However, no IVM medium is superior to other media. You could choose an IVF media as a convenient basic media such as blastocyst media (61, 63). Serum albumin and gonadotropins are typically supplemented to the IVM medium. After Day 1 (24–30 h) to Day 2 (48 h) culture, matured oocytes are cryopreserved using vitrification method or fertilized with partner sperm.

Recently, efforts have been made to try and improve human IVM culture system in order to mimic *in vivo* maturation process such as the introduction of 3-D culture systems (57, 58, 64), using C-Type Natriuretic Peptide (CNP) to keep gap junction for a certain time before starting oocyte maturation *in vitro* (54), and adding EGF-like growth factors (amphiregulin and epiregulin) (65) or oocyte secreting factors (GDF-9 and BMP-15) (66, 67) to culture medium.

In cases of cryopreservation of IVM embryos, historically, intracytoplasmic sperm injection (ICSI) has been used to inseminate the oocytes matured *in vitro* in order to increase the chances of fertilization due to potential zona pellucida hardening (68, 69). ICSI is performed more than 1 h later after the first polar body extrusion based on a report (70). Culture conditions for fertilized embryos generated from IVM oocytes are the same as those in IVF cycles.

#### Cryopreservation of Oocytes

Cryo-injuries may occur at all phases of the cryopreservation process such as chilling injury (+15°C ~ −5°C), ice crystal formation (−5°C ~ −80°C) and facture damage (−50°C ~ −150°C) depending on the temperature (71). Therefore, it is important to know the causes and mechanisms of cryo-damage in order to develop the optimal cryopreservation method.

There are two general approaches to prevent these cryo-injuries: slow-cooling and vitrification. The major difference is initial concentration of cryoprotectants and variation in cooling procedures (71). In the slow cooling method, they use a low concentration of cryoprotectants and very slow cooling rate using specialized equipment which can control the temperatures to avoid ice crystallization formation inside the cells (71). Compared to the slow-cooling method, in the vitrification, high concentration of cryoprotectants and ultrarapid cooling are used to prevent the ice crystal formation in and out of the oocyte/embryos (71, 72). Therefore, vitrification takes only a few seconds to cool oocytes or embryos after exposing them to cryoprotectants, and does not require expensive specialized equipment. Recently, many cryo-devices as a carrier have been used to increase cooling and warming rates resulting in significant increases in the success rate of human oocyte vitrification (71, 72).

Since the slow-freezing method is inefficient and inconstant, vitrification has gradually replaced slow-freezing during the past decade as a main cryopreservation method based on comparable success rates with fresh oocytes in IVF program (12, 73, 74). In addition, the vitrification method has also been applied to cryopreserve oocytes or embryos generated from IVM program (75).

### IVM Program for Fertility Preservation

Some advantages to cryopreserve oocytes generated from IVM program for fertility preservation of cancer patients are as follows;

1. Eliminating costly drugs and frequent monitoring.
2. Since IVM treatment does not need high gonadotropin stimulation, it takes no more than 48 h from the decision to perform oocyte retrieval (76). However, a stimulated IVF cycle requires more days until oocyte retrieval even though stimulation starts any phase of menstrual cycles. Therefore, when patients cannot delay chemotherapy IVM treatment may be a good option (Figure 1).
3. Avoiding the use of hormones in cancer patients with hormone-sensitive tumors. As mentioned previously, *in vivo* matured oocytes obtained from IVF cycles can be easily cryopreserved using vitrification methods with high success rates for fertility preservation. However, in some patients who have some special medical conditions or certain cancers, such as hormone-sensitive cancers, the high levels of estrogen during gonadotropin hyperstimulation are worrisome to both physicians and patients, even though several modified ovarian stimulation protocols with antiestrogen therapy have been developed (77–80). At McGill, around 70% of women who had frozen oocytes/embryos using IVM program for fertility preservation were patients with breast cancer (81).
4. Retrieving oocytes at any phase of the menstrual cycle, even in the luteal phase, without affecting the quantity and quality of the immature oocytes (22, 82). In cancer patients who are subject to time limits, immature oocyte retrieval in the luteal phase can be considered before cancer treatment in order to maximize the possibility of fertility preservation. According to our data, we did not find any significant differences in the number of oocytes retrieved, maturation and fertilization rates, or total number of available oocytes and embryos to vitrify when immature oocyte retrieval was performed during the early-, late- follicular phase compared with the luteal phase of the cycle for fertility preservation (22). There is evidence that pregnancy has occurred after donation of immature oocytes, which had been exposed to high progesterone level, retrieved from ovaries during cesarean section (83). In IVF programs, it has already been demonstrated that there is no difference of the number of oocytes collected, fertilization, their embryo developmental potential and clinical outcomes between oocytes generated from stimulations started during any phase of the menstrual cycle (84, 85).
5. The ability to harvest immature oocytes from ovarian biopsy specimens (86). Sometimes, several visible antral follicles are present on ovarian tissue biopsied for cryopreservation for fertility preservation. In this case, retrieving immature oocytes from the follicles using a syringe is an additional benefit to maximize fertility preservation (24, 86, 87). After applying this strategy, actually, a few successful live birth cases have been reported after cryopreservation of IVM embryos obtained from the ovarian tissue of cancer patients (88, 89). In addition, immature oocytes can be aspirated from the ovaries during caesarian section for the women who have cancer during pregnancy for fertility preservation (90, 91).
6. Combined *in vitro* growth (IVG) of isolated small follicles and *in vitro* maturation of the immature oocytes.

As mentioned previously, there is the risk of reintroducing malignant cells at transplantation of cryopreserved tissue (16, 17). In these cases, as an alternative, follicle isolation, *in vitro* growth (IVG) of the follicles and *in vitro* maturation of the immature oocytes would be another strategy to minimize the risk. Ideally, it is important to culture from primordial follicle stages. However, it is still challenging to establish the IVG culture system from the primordial follicle stages with no success in humans yet (92). On the other hand, M-II oocyte production has successfully been reported after combined IVG of secondary follicles isolated from fresh ovarian tissues and IVM of the immature oocytes. According to literature, human GV-stage oocytes have 3 different stages (93) and early antral follicle stage of GV-oocytes have lower maturation rate *in vitro*. Therefore, another option to preserve more mature oocytes would be *in vitro* growth (IVG) of secondary /early antral follicles isolated from ovarian tissue until meiotically competent GV oocytes are achieved, removed from the follicle and *in vitro* matured (92).

Theoretically, there are two approaches for preserving immature oocytes: oocyte cryopreservation at the mature stage (after IVM) and oocyte cryopreservation at the GV- stage (before IVM). The first successful pregnancy and live birth using immature human oocytes was after freezing at GV-stage oocytes (27), but further successful cases were from cryopreservation at MII-stage oocytes after IVM (28).

Some advantages and disadvantages in both approaches have been reported.

Table 1 shows characteristics of GV-stage and MII- stage oocytes. MII-stage oocyte has chromosomes attached with meiotic spindles. These microtubules are considered prone to damage at low temperature (chilling injuries), and the spindle dysfunction increases the risk of aneuploidy caused by chromosome misalignment. There is therefore a potential risk of affecting the meiotic spindle during vitrification and warming procedures in the MII-stage oocytes. It is possible that GV-stage oocytes are more stable when cryopreserved than MII-stage of oocytes, since they does not have temperature-sensitive meiotic spindle and their nuclear membrane could protect prophase-I chromatin genetic material during cryopreservation. In addition, the first successful live birth with immature oocytes was after freezing at GV-stage oocytes (27). Paradoxically, however, increasing chromosome and spindle abnormalities were observed in MII-stage oocytes matured *in vitro* after cryopreservation at GV-stage oocytes using slow-cooling method (8). This is probably due to lower cell membrane stability in immature oocytes associated with membrane lipid phase transition temperature (94). In addition, some data showed that aneuploidy rate was not increased after vitrification of human oocytes matured *in vivo* or *in vitro* (95, 96).

**Table 1:** Different characteristics of oocyte between GV- and MII- stage.

**Characteristics**	**GV-stage**	**MII-stage**
Meiotic spindle	–	+
Nuclear membrane	+	–
Cell membrane lipid stability	Lower	Higher
Cumulus cells	+	–
- cell size		
- gap junction		

While MII-stage oocytes do not require CC to be attached to the oocyte at cryopreservation, GV stage oocytes need CC in order to get nutrients and regulatory molecules through gap junctions after thawing/warming, which are needed for oocyte maturation and further embryo development (97). However, there are two problems when trying to preserve these physiological interactions. The first, since there is a difference in the surface/volume ratio between oocyte and CC, the optimal exposure times and concentrations of cryoprotectants for equilibration are likely to be different and so it is difficult to make optimal cryopreservation conditions for both cell types. The other problem is that immersion GV-stage oocytes with CC in hypertonic cryoprotectants cause cell shrinkage of both oocyte and CC leading to disruptions in the gap junction between the oocyte and CC during cryopreservation (98).

### Studies Concerning Human IVM Oocyte Vitrification

Several studies have been published in the field of IVM oocyte vitrification.

Chung et al. (95) compared survival and embryo development after vitrification of different stages of oocytes retrieved from unstimulated or stimulated ovaries. The immature oocytes collected from unstimulated ovaries were vitrified either at GV- or MII-stages after IVM. The immature oocytes retrieved from stimulated ovaries (conventional IVF cycles) were vitrified at GV-, GVBD- (MI-), or MII-stages after IVM for different time periods. After warming, they compared survival, maturation and embryo developmental competence in the oocytes vitrified at different stages. Although the number of samples was too small to get statistical significance in both sources of immature oocytes, in the immature oocytes collected from unstimulated ovaries, there were no differences in the rates of IVM, survival and blastocyst development between oocytes vitrified at GV- (before) (63.2, 63, 43%) and MII- (after) stage (69.6, 56, 40%), respectively. However, in the immature oocytes collected from conventional IVF cycles, the rates of survival, fertilization and blastocysts development were better in the group where oocytes were vitrified at M-II stage (100, 83.3, 40%) compared to GV-(65, 66.7, 33.3%) or GVBD-stage (64.2, 55.6, 20%), respectively. In addition, IVM was affected after vitrifying the oocytes at GV- or GVBD-stage. The blastocysts produced from each group had normal chromosome competency. This study showed different embryological results depending on the source of immature oocytes obtained from either stimulated or unstimulated ovaries.

Cao et al. (99) compared embryological aspects between oocytes before and after IVM of immature oocytes obtained in IVM cycles. This study included PCOS patients who were given clomiphene and hMG for 5 days and primed with hCG when leading follicles reached 8 to 10 mm. Egg collection was performed 36 h after administration of 10,000 IU hCG. The retrieved oocytes were divided into three groups; control fresh *in vitro* matured M-II, before IVM (GV stage) and after IVM (M-II stage) groups. They did not observe any difference in the survival rate between oocytes vitrified at GV- and MII-stages (85.4% vs. 86.1%). However, there was a significant difference in the IVM rates between immature oocytes with and without vitrification. Higher IVM was obtained in the oocyte without vitrification (85.4%) than that of vitrified immature oocytes (50.8%). Better quality embryos at the cleavage stage were produced from the group where oocytes were vitrified at M-II stage (33.3%) than the group where oocytes were vitrified at GV-stage (12.2%). In both groups, the embryo developmental competence was lower than that of fresh IVM oocytes (49.3% of good quality and 46.3% of blastocysts developed). Therefore, from this study it would appear that vitrifying oocytes at the M-II stage, after IVM, improves the chance of success compared with freezing them at the GV stage and *in vitro* maturing them after warming. However, they did not assess the oocyte maturity at the time of collection. As mentioned before, IVM cycles related with hCG priming can induce oocyte maturation *in vivo* and it would be possible to collect some M-II- and GVBD-stage oocytes even from follicles <10 mm in diameter. According to the published literature, over 50% of oocytes are retrieved from follicles sized 10 mm diameter at oocyte retrieval, even in conventional IVF (100). Therefore, this study needs to be confirmed.

Conversely, Wu et al. (101) reported embryo developmental competence as being similar between oocytes with and without vitrification in GV-stage oocytes collected from unstimulated ovaries, even though survival rate was low after vitrification (59.0%). Similarly, Al-khtib et al. (102) observed that the IVM rate of GV-stage oocytes retrieved from stimulated ovaries was similar with and without vitrification (75.5% vs. 70.8%).

Chang et al. (103) compared survival and embryo development after vitrification between oocytes matured *in vivo* and *in vitro* in oocyte donation (OD) IVF cycles and reported that there was no difference in survival rates. However, embryo development was better in embryos generated from oocytes matured *in vivo* than *in vitro*. Nevertheless, it is difficult to know whether the lower embryo development of the oocytes matured *in vitro* was because of oocyte innate characteristics or because of the vitrification and warming process. In general, the embryos generated from immature oocytes in conventional IVF cycles have less developmental potential than sibling embryos produced from *in vivo* matured oocytes.

Fasano et al. (104) used GV- and GVBD (MI)-stage oocytes obtained from ICSI cycles in order to compare oocyte survival, maturation and embryo developmental competence between oocytes vitrified before and after IVM of immature oocytes. Although the survival rate of oocytes after warming was similar (86.9% vs. 84.5%), maturation rate was significantly higher in the oocytes matured *in vitro* before vitrification (46%) than that of oocytes vitrified at immature stages before IVM (23.8%) (*P* < 0.01). After insemination, the fertilization and embryo developmental rates were not significantly different, but no blastocysts were produced in both groups. Accordingly, vitrifying oocytes after IVM was more efficient than that of IVM after vitrification to get more MII oocytes to inseminate. This is because vitrifying oocytes at GV-stage affect their IVM capability negatively after warming.

However, Molina et al. (105) presented different results showing higher IVM rate after vitrifying oocytes at GV-stage than that of control fresh IVM oocytes obtained from stimulated IVF cycles. Although activation rate was lower, activated oocytes from the group where oocytes were vitrification at GV-stage developed more embryos compared to the group where oocytes were vitrified at MII after IVM. Therefore, vitrifying GV-stage oocytes seemed to be better than that of MII-stage in terms of IVM rate and embryo development.

Song et al. (106) were also using GV- and GVBD (MI)-stage oocytes generated from IVF cycles. Control was oocyte matured *in vivo* without vitrification and vitrification groups were where vitrification was performed before or after IVM of immature oocytes. After warming, cleavage rate was significantly higher in the oocytes vitrified at MII-stage after IVM than the group where oocytes vitrified at immature stage before IVM (*P* < 0.05). However, there were no statistical differences in fertilization, embryo developmental competence and aneuploidy rate in the three groups. This study indicates that oocytes at MII-stage after IVM were more suitable to vitrify than oocytes at immature stages.

Kasapi et al. (107) used GV oocytes collected from stimulated donation cycles. Oocytes were either vitrified at the GV stage or at MII-stage after IVM. Control group was oocytes matured after IVM without vitrification. There were no significant differences in the survival rate or incidence of normal spindle/chromosome configurations in oocytes matured *in vitro* before or after vitrification. A higher incidence of normal spindle/chromosome configurations existed in the fresh IVM oocytes. However, a significantly higher maturation rate was obtained in the group where oocytes were vitrified after IVM (82.9%) compared to oocytes vitrified at GV stage (51%). Their study also demonstrated that vitrification of *in vitro* matured MII-oocytes obtained from stimulated cycles was more efficient than GV oocytes vitrification.

Table 2 summarizes these 9 studies. There were differences among the groups in terms of devices, solution, source of immature oocytes, and IVM culture system. In addition, sample sizes in most of studies were not enough. Therefore, it appears difficult to draw general conclusions from these studies. However, in stimulated cycles, it seems that vitrifying oocytes at MII-stage after IVM is better than at GV-stage in terms of IVM rate after warming, even though Molina et al. (105) reported a reverse result. This needs to be verified in oocytes obtained from unstimulated cycles since two studies in Table 2 showed no difference of IVM rate with and without vitrification at the GV stage. Nevertheless, they were overall similar in the rates of survival, fertilization and embryo developmental potential. However, the studies show that vitrification/warming of oocytes either at GV- or MII-stages severely affect their fertilization and embryo developmental potential compared to fresh oocytes. This is evidence that oocytes obtained from IVM are more fragile for the cryopreservation compared to *in vivo* matured oocytes.

**Table 2:** Summary of IVM oocyte vitrification studies.

**References**	**Carrier**	**Cryo-solution**	**Source of immature oocytes**	**IVM media**	**Affect on IVM after vitrifying at immature stage**	**Survival rate**	**2 PN**	**Embryo development**
Chung et al. (95)	EM-grid	EG	Unstimulated IVF	TCM-199 + PMSG + HCG	No Yes	GV≈MII GV < MII	GV≈MII GV < MII	GV≈MII GV < MII
Cao et al. (99)	Cryoleaf	EG +PROH	CC+HMG	TCM-199 + FSH + HCG	Yes	GV≈MII	GV≈MII	GV < MII
Wu et al. (101)	EM-grid	EG	Unstimulated	Ham's F10 + HMG	No	59%	Frozen ≈ Fresh	Frozen ≈ Fresh
Al-khtib et al. (102)	High-security Straw	EG +PROH	IVF	Medicult IVM + FSH + HCG	No	55.4%	NA	NA
Chang et al. (103)	Cryotop	EG +DMSO	IVF	Fertilization Medium (SAGE)	–	*In vivo* MII ≈*in vitro* MII	*In vivo* MII > *in vitro* MII	*In vivo* MII > *in vitro* MII
Fasano et al. (104)	High-security Straw	EG +DMSO	IVF	SAGE IVM medium + FSH + LH	Yes	GV/MI < MII	GV/MI ≈ MII	GV/MI ≈ MII
Molina et al. (105)	Cryo-tip	EG +DMSO	IVF	Medicult IVF medium + FSH + LH	No (Improve)	GV≈MII	GV < MII (by activation)	GV > MII (by activation)
Song et al. (106)	Cryoleaf	EG +PROH	IVF	SAGE IVM medium + FSH + LH	Yes	GV/MI≈MII	GV/MI < MII	GV/MI > MII
Kasapi et al. (107)	Closed carrier system (VitriSafe, VitriMed, Austria)	EG +DMSO	IVF	SAGE IVM medium + FSH + LH	Yes	GV≈MII	–	–

Again, there were different characteristics of immature oocytes depending on their source of origin and the method of vitrification. As shown in the Cao et al. (99) study, different results could be obtained in immature oocytes retrieved from different sources of oocytes with and without gonadotropin stimulation. Most of IVM cases for cancer patients are from unstimulated ovaries. Therefore, further investigations with immature oocytes retrieved from unstimulated ovaries are needed to improve fertility preservation options of IVM.

Based on overall results from these studies, we vitrified MII-stage oocytes after IVM in unstimulated IVM cycles at McGill (108). We recruited patients for IVF and IVM oocyte vitrification studies and compared the two groups. There were no differences in patient characteristics between two groups. Compared with *in vivo* MII-stage oocytes generated from IVF cycles, MII-stage oocytes obtained after IVM of immature oocytes in unstimulated regular IVM cycles had significantly lower survival (81.4% vs. 67.5%) and fertilization (75.6% vs. 64.2%) rates after vitrification (*P* < 0.05). In addition, implantation (19.1% vs. 9.6%), clinical pregnancy (44.7% vs. 20.0%), and live birth (39.5% vs. 20%) rates were lower in IVM-oocyte vitrification groups, but it was not statistically significant since the number of samples was not sufficient. From this study, it is difficult to determine whether the lower embryological and clinical outcomes in the oocytes from our IVM program is due to the low quality of IVM oocytes, our vitrification procedure, or both since there was no fresh IVM oocytes control group included in the study. In addition, no reproductive potential has been reported to compare fresh and vitrified IVM oocytes. Therefore, we analyzed embryological and clinical outcome of IVM oocyte vitrification with that of fresh IVM cycles performed during the same period (28).

We included 267 patients for the study, 56 patients were for IVM oocyte vitrification group and 219 patients were fresh IVM group during the same period. Oocyte maturation rates were similar between the groups. Survival rates of IVM oocytes after performing vitrification/warming was 59.8%. The rates of fertilization and embryo cleavage in the vitrified IVM oocytes (58%, 72%) were significantly lower compared to fresh IVM oocytes (72%, 90%) (*P* < 0.01). Clinical pregnancy (10.7% vs. 36.1%) and live birth rates (8.9% vs. 25.9%) were also significantly lower in the group where IVM oocytes were vitrified than those in the group where IVM oocytes were fresh (*P* = 0.005 and *P* < 0.001, respectively). Therefore, reproductive potential was negatively affected after vitrification of IVM oocytes. This implies that vitrification/warming itself could also induce some detrimental effects on IVM oocytes.

Given *in vitro* matured oocytes may never be equivalent to *in vivo* matured oocytes, it is necessary to modify vitrification methods/process appropriately for oocytes retrieved from the IVM programs, to improve survival and embryo developmental rates of IVM oocytes. There is evidence that *in vivo* matured oocytes collected from advanced aged women (>35 years old) also had lower survival rates (82.4%) after vitrification than those of oocytes collected from younger women (≤35 years old) (94.6%) (109). It indicates that oocyte quality is one of the main factors related with survival rates as well as clinical outcomes.

Actually, present vitrification methods have been adapted to use good quality *in vivo* matured oocytes from young women. Therefore, studies to improve survival and further embryological developmental competence of the oocytes retrieved from IVM program are urgently required in order to successfully apply them to IVM fertility preservation program for cancer patients.

## Conclusions

Immature oocytes may be the answer for fertility preservation in the long term. Healthy live births can be achieved from the combination of IVM oocytes and vitrification even though no live births have been reported using cryopreserved oocytes generated from IVM program of cancer patients. The efficiency of IVM oocyte cryopreservation is still low and requires further improvements in IVM- or/and Cryo-technology. Different strategies may be implemented in order to improve the results such as improving oocyte quality by optimizing the *in vitro* culture conditions of immature oocytes and/or establish a more refined vitrification/warming procedure which can adjust to cellular properties of oocytes before/after IVM. Improved understanding of mechanisms regulating IVM and developing subsequent optimal IVM medium may give rise to more refined vitrification methods for oocytes before/after IVM. This will in turn help maintain the same oocyte quality before and after vitrification, resulting in improved quality of embryos and pregnancy rates.

## Author Contributions

W-YS's role included study design, data collection, statistical analysis, and manuscript writing. SH was involved in data collection and manuscript writing and review. YC's role was data collection and statistical analysis. MD was involved in study design and manuscript writing. WB's role included study design, manuscript writing, and review.

### Conflict of Interest Statement

The authors declare that the research was conducted in the absence of any commercial or financial relationships that could be construed as a potential conflict of interest.
